# Transcription factor binding sites are highly enriched within microRNA precursor sequences

**DOI:** 10.1186/1745-6150-6-61

**Published:** 2011-12-02

**Authors:** Jittima Piriyapongsa, I King Jordan, Andrew B Conley, Tom Ronan, Neil R Smalheiser

**Affiliations:** 1 Genome Institute, National Center for Genetic Engineering and Biotechnology, 113 Thailand Science Park, Klong1, Klong Luang, Pathumthani 12120, Thailand; 2 School of Biology, Georgia Institute of Technology, Atlanta, Georgia 30332 USA; 3 Department of Bioengineering, University of Illinois at Chicago, Chicago, Illinois, USA; 4 Department of Psychiatry, University of Illinois at Chicago, UIC Psychiatric Institute MC912, 1601 W. Taylor Street, Chicago, IL 60612 USA

**Keywords:** Transcription factors, microRNA biogenesis, drosha

## Abstract

**Background:**

Transcription factors are thought to regulate the transcription of microRNA genes in a manner similar to that of protein-coding genes; that is, by binding to conventional transcription factor binding site DNA sequences located in or near promoter regions that lie upstream of the microRNA genes. However, in the course of analyzing the genomics of human microRNA genes, we noticed that annotated transcription factor binding sites commonly lie within 70- to 110-nt long microRNA small hairpin precursor sequences.

**Results:**

We report that about 45% of all human small hairpin microRNA (pre-miR) sequences contain at least one predicted transcription factor binding site motif that is conserved across human, mouse and rat, and this rises to over 75% if one excludes primate-specific pre-miRs. The association is robust and has extremely strong statistical significance; it affects both intergenic and intronic pre-miRs and both isolated and clustered microRNA genes. We also confirmed and extended this finding using a separate analysis that examined all human pre-miR sequences regardless of conservation across species.

**Conclusions:**

The transcription factor binding sites localized within small hairpin microRNA precursor sequences may possibly regulate their transcription. Transcription factors may also possibly bind directly to nascent primary microRNA gene transcripts or small hairpin microRNA precursors and regulate their processing.

**Reviewers:**

This article was reviewed by Guillaume Bourque (nominated by Jerzy Jurka), Dmitri Pervouchine (nominated by Mikhail Gelfand), and Yuriy Gusev.

## Background

MicroRNAs are important post-transcriptional regulators of gene expression [1]. However, they do not work in isolation, but rather act in concert with other classes of regulatory proteins. In particular, transcription factors, microRNAs and their respective targets form interconnected feedback and feedforward circuits [2]. Transcription factors are thought to regulate the transcription of microRNA genes in a pol II dependent manner similar to that of protein-coding genes; that is, by binding to conventional transcription factor binding site sequences (TFBS) located in or near promoter regions that lie upstream of the microRNA genes [3,4]. In the course of analyzing the genomics of human microRNA genes using the UCSC Genome Browser, we noticed that annotated transcription factor binding sites commonly lie within 70- to 110-nt long microRNA small hairpin precursor (pre-miR) sequences. In this short report, we characterize this association in detail and discuss several possible explanations for this surprising phenomenon.

## Results and discussion

**Figure 1:**
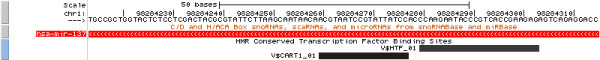
**Alignment of Conserved TFBS track and Sno-miRNA track**. Shown are the Conserved TFBS and sno/miRNA tracks from the UCSC Genome Browser in the region corresponding to the small hairpin microRNA precursor encoding hsa-mir-137. The Conserved TFBS track is shown at its default setting (using stringent criteria to display conserved TFBS having z-score ≥ 2.33).

The TFBS Conserved track is available for display in the UCSC Genome Browser [5], (March 2006, NCBI36/hg18 assembly) and indicates the computed location and score of 398 transcription factor binding site motifs included in the TRANSFAC database. These are consensus motif sequences, generally 6- to 14-nt in length, that are conserved in the human/mouse/rat alignment. One can selectively view datasets that satisfy different levels of statistical significance (ranging from z-score ≥ 1.64, which is equivalent to a significance value of p < 0.05 using one-tailed t-tests, up to z-score ≥ 2.33, which is equivalent to a significance value of p < 0.01). If one views the TFBS Conserved track in juxtaposition to the sno/miRNA track (which indicates the positions of 70- to 110-nt long small hairpin precursor (pre-miR) sequences of microRNA genes taken from miRBase), it can readily be appreciated that many pre-miRs contain one or more conserved TFBS (Figure 1).

**Figure 2:**
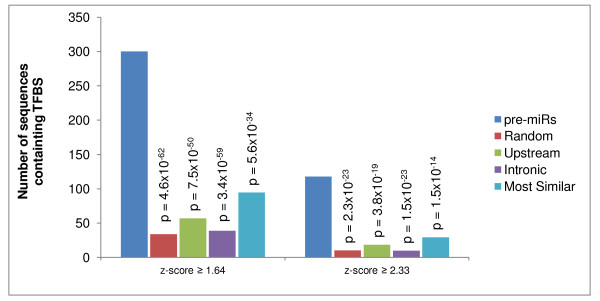
**miRNA-TFBS association compared against four types of negative control sequences**. We compared the observed prevalence of TFBS motifs in pre-miR sequences against four types of negative control datasets. For each human pre-miR, 1,000 sequences having the same length as the pre-miR were selected from the human genome according to one of 4 different rules and were examined for the presence of TFBS motifs. In the first negative control dataset, for each pre-miR, 1,000 sequences were chosen *randomly *in the genome and the average number of sequences containing at least one TFBS motif was scored. In the second negative control dataset, for each pre-miR, negative control sequences were chosen within 5 kb *upstream *of annotated genes. In the third case, negative control sequences were chosen within *introns*. In the fourth dataset, negative control sequences were chosen randomly and filtered to retain those which are most similar to pre-miRs in their cross-species conservation and dinucleotide composition (see Methods). Statistical significance was assessed by chi-square test.

Across all 715 human pre-miRs, there are 300 cases in which at least one predicted conserved TFBS (at p < 0.05) is fully contained within a pre-miR sequence, and in an additional 27 cases the TFBS partially overlaps with a pre-miR (Figure 1, Figure 2). In fact, if one removes from consideration the 297 miRNAs that are primate-specific [6] (and hence cannot contain conserved regions), over 75% of the remaining pre-miRs contain at least one conserved TFBS. This association cannot be ascribed to chance, since the statistical significance value is essentially zero (i.e., less than p = 1 × 10^-10^). Moreover, the p-value remains near zero when one considers only high-confidence TFBS with a z-score ≥ 2.33 (Figure 2). The association is retained whether one compares pre-miRs to negative control sequences chosen randomly in the genome; to sequences that lie within 5 kb upstream of annotated protein-coding genes; to sequences that lie within introns; or to random genomic sequences filtered to include only those that most closely resemble pre-miRNA sequences in terms of sequence conservation, mono-nucleotide content and di-nucleotide composition (see Methods) (Figure 2).

The foregoing analysis based on the TFBS Conserved track can only shed light on TFBS motif hits upon sequences that are conserved across mouse, rat and human. Therefore, we also carried out an independent analysis in which all human pre-miR sequences were scored for the presence of TFBS motifs at all points along the entire pre-miR sequence, without regard to whether the sites were conserved or not. In this case, each pre-miR sequence was randomly permuted 1,000 times (maintaining dinucleotide composition), and each permuted sequence was scored for TFBS motifs.

**Figure 3:**
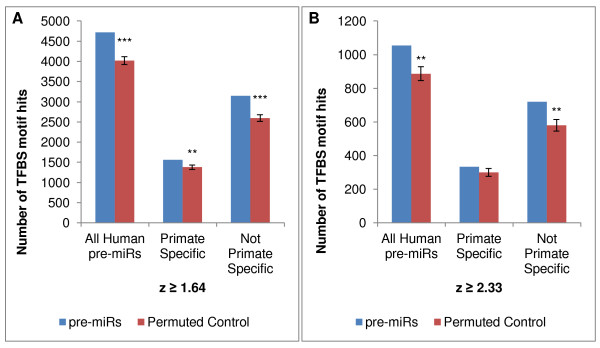
**TFBS motif hits in pre-miRs as compared to permuted negative control sets**. Human pre-miR sequences were scanned directly for predicted transcription factor binding sites using matrices from the TRANSFAC 7.0 Public Database at the indicated stringency (z-score ≥ 1.64 or 2.33, see Methods). For each pre-miR, a randomized negative control set was created consisting of 1,000 iterations of permuted sequences matched for length and dinucleotide frequency. Each of the randomized sequences was then scanned for TRANSFAC motif hits in the same manner. **significant at p < 3 × 10^-5^. ***p < 2 × 10^-12^.

As shown in Figure 3, the observed number of TFBS hits upon all human pre-miRs was significantly greater than the average number of hits upon their randomized permuted counterparts, both at z-scores ≥ 1.64 and ≥ 2.33. The effect was extremely significant for miRNAs that are not primate-specific. In contrast, the subset of primate-specific miRNAs showed a lesser over-representation of hits satisfying z-score ≥ 1.64 that was not significant for hits at the more stringent criterion of z ≥ 2.33 (Figure 3). These data confirm and extend the results described above using the Conserved TFBS track, and verify that the association of TFBS hits is not an artifact of examining only conserved pre-miR sequences.

### Characterizing the pre-miRs that do vs. do not contain conserved TFBS motifs

We analyzed further the set of 118 human pre-miRs that completely contained at least one conserved TFBS using stringent criteria (z-score ≥ 2.33, p < 0.01; Figure 2, Additional Files 1 and 2). TFBS were associated with both intergenic and intronic pre-miRs and with both isolated and clustered microRNA genes (Table 1). Many of the best-studied miRNAs contained TFBS (e.g., mir-200a, b, c; mir-125a; let-7b), including those that have wide tissue expression patterns (e.g. mir-16-2) and others enriched in specific organs such as brain (mir-124-1,2) or liver (mir-122) [7]. The association of TFBS with pre-miRs affects a wide range of miRNAs with known functions, including many of those involved in cancer pathways [8] and those involved in neural disorders [9].

**Table 1:** Genomic location of pre-miRs containing conserved TFBS (z-score ≥ 2.33).

(A)	
**Genomic region**	**Number of pre-miRs**

Intergenic	49

Intron	48

Exon	10

Exon-intron	11

Total	118

**(B)**	

**Location of miRNA**	**Number of pre-miRs**

In cluster	60

Not in cluster	58

Total	118

"In cluster" means that there is at least one other pre-miR encoded within 10 kb of its location.

The majority of pre-miRs associated with TFBS are highly conserved across multiple vertebrate classes and are not simply mammalian-specific (Table 2). Moreover, pre-miRNA sequences containing TFBS show significantly higher conservation values (i.e., fewer evolutionary changes) than pre-miRs not associated with TFBS, even when one only compares nucleotide changes occurring between the macaque and human genomes: Using the data provided in Qiu et al [10], the mean evolutionary rates for the TFBS group vs. without-TFBS group are 0.059 vs. 0.111, which are significantly different (p = 0.0019). As a group, the pre-miRNA sequences containing TFBS have higher average expression levels across human tissues than the ones lacking TFBS (Table 3). Moreover, only 1 of the 118 pre-miRs that contained TFBS contained any annotated transposable element sequences, vs. 26% of pre-miRs that did not (χ^2 ^= 36.1, p = 1.9 × 10^-9^).

**Table 2:** Conservation profile of pre-miRs containing conserved TFBS (z-score ≥ 2.33).

		Lineage		
		
	Primate	Mammal	Vertebrate	Row total
Pre-miRs containing TFBS	9	40	69	118

Pre-miRs lacking TFBS	316	146	119	581

Column total	325	186	188	699

"Primate" lineage indicated here includes miRNAs that have pre-miR homologues in non-primate species but which do not match the mature human miRNA sequence exactly. The lineage distribution is extremely different between pre-miRs that contain vs. those that lack TFBS (χ^2 ^= 101.59, p = 0).

**Table 3:** Average expression across human tissues of pre-miRs containing conserved TFBS (z-score ≥ 2.33).

	Expression level				Row total
	**High**	**Intermediate**	**Low**	**Very low**	

pre-miRs containing TFBS	30	21	14	10	75

pre-miRs lacking TFBS	55	74	81	91	301

Column total	85	95	95	101	376

Pre-miRs that contain TFBS tended to have higher expression than those lacking TFBS (χ^2 ^= 20.82, p = 0.0001).

Conserved TFBS were much more strongly associated with pre-miR sequences than with their immediately flanking upstream and downstream regions: Whereas 118 pre-miRs contained conserved TFBS, only 64 pre-miRs contained TFBS in their immediate upstream or downstream regions combined (having twice the total length as the pre-miR region) (Table 4). Among pre-miRs that contained a conserved TFBS, 19% of cases expressed one or more TFBS in its flanking regions as well, in contrast to only 7% of other pre-miRs. Thus, pre-miR sequences contained significantly more TFBS than their flanking regions, though the flanking regions of TFBS-containing pre-miRs also had more TFBS than the flanking regions of pre-miRs that lacked TFBS. These data extend an earlier observation [11] that TFBS are enriched in regions immediately flanking pre-miR hairpins, relative to regions further upstream or downstream.

**Table 4:** Distribution of TFBS in pre-miRs and their flanking regions.

	pre-miRs with TFBS in flanking	pre-miRs without TFBS in flanking
pre-miRs containing TFBS	22	96

pre-miRs lacking TFBS	42	540

For a given pre-miR, its flanking region was defined as comprising the region upstream (distance equal to the pre-miR) and downstream (distance equal to the pre-miR). Given that a pre-miR contains one or more TFBS, it is more likely to have TFBS in its flanking regions as well. Conversely, TFBS were also more common in flanking regions of pre-miRs that contained TFBS (χ^2 ^= 15.423, p = 0.00008).

**Figure 4:**
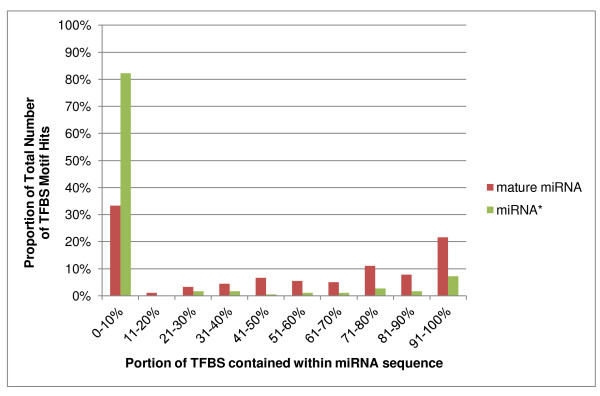
**TFBS motifs tend to overlap with mature miRNA sequences**. For all human pre-miRs, each TFBS motif hit was tabulated in terms of how fully it overlapped with the mature miRNA sequence or miRNA* sequence. 100% means that a TFBS is completely contained inside the miRNA or miRNA* mature sequence.

Conserved TFBS were equally associated with 5'- and 3'- halves of the pre-miR sequences. Using miRBase assignments to identify mature miRNA and minor (miRNA*) sequences, we observed that conserved TFBS were found in all regions within the pre-miRs including the loop and near the base of the stem (Additional File 2), but showed a predominant association with mature miRNA sequences. Two-thirds of the TFBS motifs overlapped the mature miRNA, and half overlapped the mature miRNA sequence by 11 or more bases. However, the TFBS motifs were usually not precisely co-located with, or contained entirely within, the annotated mature miRNA sequence (Figure 4 and Additional File 2). Conserved TFBS showed much less overlap with miRNA* sequences (Figure 4 and Additional File 2).

A possible explanation for the presence of TFBS within pre-miRs is that they might be involved in regulating transcription of nearby downstream genes. On the one hand, as shown in Table 5, about 25% of TFBS-associated pre-miRs lie upstream within 5 kb of another annotated gene, compared to 15% of pre-miRs lacking TFBS. As well, about 25% of pre-miRs that lie upstream of another annotated gene contain at least one TFBS, in contrast to 15% of the other pre-miRs. These differences are significant (p = 0.008), and demonstrate that the incidence of TFBS within pre-miRs is a function of their genomic positioning. However, the great majority of TFBS-associated pre-miRs do not have a close or obvious relationship to nearby downstream genes.

**Table 5:** Distribution of pre-miRs containing TFBS (z-score ≥ 2.33) relative to nearby downstream genes.

	pre-miRs containing TFBS	pre-miRs lacking TFBS
pre-miRs is upstream	29	85

pre-miRs is not upstream	89	496

Pre-miRs containing TFBS are more likely to reside ≤ 5 kb upstream of nearby genes, and pre-miRs that lie upstream of nearby genes are more likely to contain TFBS (χ^2 ^= 7.109, p = 0.008).

**Figure 5:**
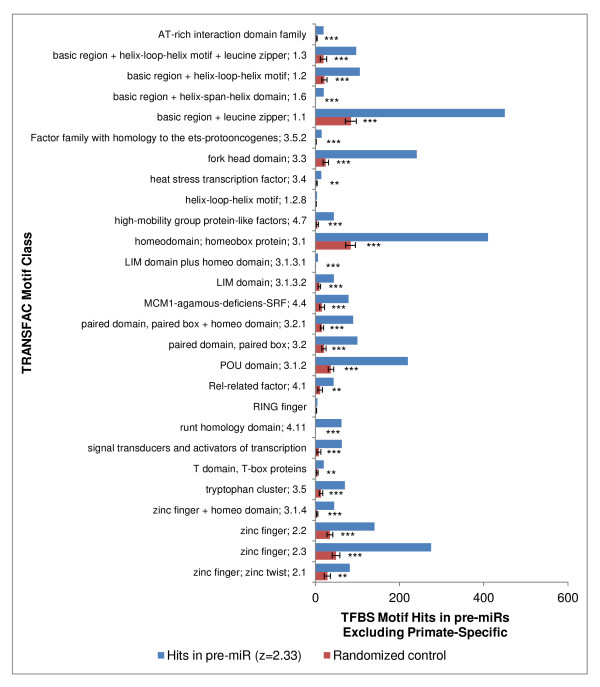
**TFBS motif hits on human pre-miRs tabulated by motif class, excluding those that are primate specific**. TRANSFAC motif classes are shown, tabulating hits upon human pre-miRs excluding primate specific pre-miRs. Hits in the set of pre-miRs are compared to hits observed within the corresponding randomized negative control sets (see figure 3 and Methods). *significant at p < 0.01. **p < 1 × 10^-7^. ***p < 1 × 10^-14^.

**Figure 6:**
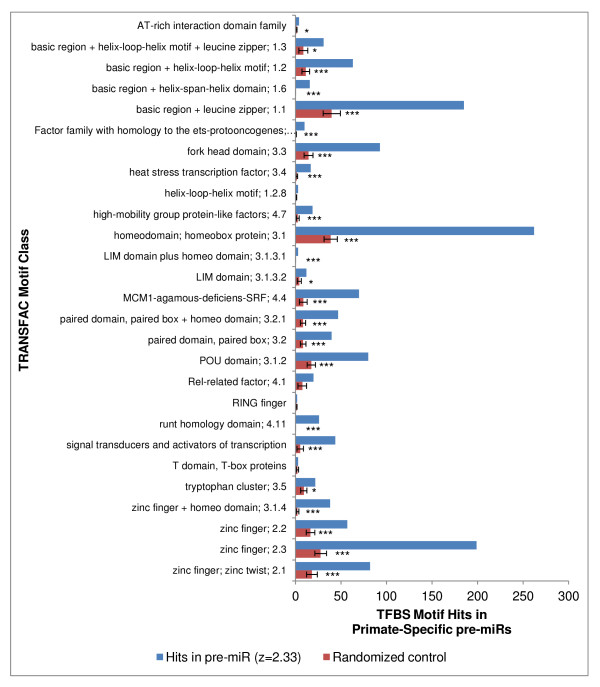
**TFBS motif hits on human pre-miRs that are primate specific**. Same as figure 5 but showing results for primate specific pre-miRs.

Many different TFBS motifs were significantly associated with pre-miR sequences, with no single one being predominant. When we examined all TFBS motif hits (not just the conserved hits) upon all pre-miRs vs. their permuted counterparts, all of the 27 different motif classes were over-represented at high stringency (z ≥ 2.33) among pre-miRs that are not primate-specific (Figure 5), and 24 out of the 27 motif classes were significantly over-represented upon primate-specific pre-miRs (Figure 6). In both subgroups, the three most prevalent motif classes were "basic region + leucine zipper", "homeodomain", and "zinc finger 2.3" (Figure 5 and 6). Similar findings were also obtained when only conserved TFBS sites were analyzed (data not shown).

## Conclusions

Classically, transcription factor binding site (TFBS) motifs are thought to represent sites on DNA that bind specific transcription factors (TFs), which in turn regulate transcription of nearby genes. The present report demonstrates that small hairpin precursor (pre-miR) sequences in the human genome contain TFBS motifs with very high prevalence and with extremely high statistical significance. What might be the biological significance of this unanticipated association?

MicroRNA genes are thought to have a conventional mRNA-like system of transcriptional regulation, in which TFBS reside largely within promoter regions lying upstream of the transcriptional unit [3,4,11]. It is possible that TFBS within pre-miRs regulate transcription of nearby downstream genes, though most pre-miRs that contain TFBS do not reside within 5 kb of annotated protein-coding genes.

Alternatively, TFBS within pre-miRs might serve specifically to regulate transcription of the primary miRNA gene transcript (pri-miR) itself. It has long been noted that pre-miR constructs lacking exogenous promoters can express some residual transcriptional activity [12], and recently Song Gao et al [13] reported that pre-miR and other microRNA gene fragments contain atypical promoter elements that can drive transcription, especially in situations where the endogenous miRNA gene is expressed at low levels. Since this paper was originally submitted for publication, Zhu et al [14] have reported that human pre-miR sequences are also associated with genomic features of active promoters, namely, positioned nucleosomes, specific histone modifications and RNA polymerase II occupancy. As well, Tata et al [15] reported the existence of an intragenic enhancer and CpG island at a pre-miR located within the pluripotency associated microRNA cluster EEmiRC. These features support the idea that TFBS within pre-miRs are related to pri-miR transcription.

It is also possible that transcription factors may bind directly to the pri-miR and/or pre-miR to regulate their processing, possibly as part of (or competing with) the drosha/DGCR8/p68 complex. Indeed, the NF90 component of the NF90-NF45 complex has been shown to bind directly to the stem-loop regions of certain pri-miRs and pre-miRs where it appears to regulate binding of the drosha complex [16]. Although the binding of transcription factors to RNA has been relatively little explored, certain transcription factors have been shown to bind specific RNAs including dsRNAs [17,18]. Consistent with this view are the observations that DGCR8 can bind NF90 [19], that the RNA helicase p68 is a transcriptional coregulator and can bind transcription factors [20], and that drosha is associated with transcriptional activators such as Ewing sarcoma protein that also possesses RNA-binding domains [21]. Such a role for TFs would be congruent with the known role of other proteins such as R-Smads, KSRP, hnRNP A1 and LIN28, which bind to specific pre-miR stem or loop sequences within pri-miRs and regulate processing of subsets of miRNAs by drosha and/or dicer [22-24].

Our findings have focused on relatively short TFBS motifs that localize the TFBS with high resolution and with high confidence. However, it is likely that the association of TFBS with pre-miRs is even stronger than reported here, since our analyses did not include variant TFBS motifs or transcription factors not included in the TRANSFAC database. We hope that this bioinformatics analysis will stimulate experimental efforts to establish the biological meaning of this phenomenon.

## Methods

### Data related to the Conserved TFBS Track

For the data related to the Conserved TFBS Track of the UCSC Genome Browser, human miRNA sequences were obtained from version 13.0 (hg18, NCBI build 36) of the miRBase database [25]. The data of Conserved TFBS track, including associated TFs were downloaded from the UCSC Table Browser [26]. The Conserved TFBS track contains the location of ~3,800,000 motifs (range 6-30 nt) that are conserved across the human/mouse/rat alignment based on the score computed with version 7.0 of the TRANSFAC Matrix Database. Four types of negative control sequences were generated for the study of miRNA-TFBS association: 1) Genomic sequences chosen randomly. 2) 5000 nt upstream sequences and 3) intron sequences taken from knownGene (UCSC Genes), which is a conservative set of gene predictions based on data from RefSeq, Genbank, CCDS and UniProt. 4) Random genomic sequences filtered to obtain those that are "most similar" to pre-miRs in cross-species conservation and dinucleotide composition (see following paragraph). For each sequence type, 715 sets (one for each pre-miR) of 1,000 random sequences of the same length were produced and the co-location with TFBS sequences was determined.

To create the negative control set of "most similar" sequences for Figure 2, we created 1,000 control sets of random genomic sequences that most closely resemble pre-miRNA sequences in terms of sequence conservation, mono-nucleotide content and di-nucleotide content and analyzed the TFBS hits on these sequences that were predicted on the Conserved TFBS Track. To do this, the 715 pre-miRNA loci analyzed here were first evaluated to yield distributions of 1) sequence conservation (using the UCSC Genome Browser 17-way phastCons base-wise conservation scores), 2) mono-nucleotide composition (*i.e. *GC content) and 3) di-nucleotide composition computed as relative frequencies of the 16 possible di-nucleotides. Average and standard deviation (SD) values for each of the distributions were then computed. The human genome sequence was then analyzed to isolate negative control sequences that most closely resemble the conservation and sequence composition distributions of the pre-miRNAs. To do this, the genome sequence was partitioned into 150 bp non-overlapping windows, and for each window: 1) conservation, 2) mono-nucleotide composition and 3) di-nucleotide composition values were computed. Individual windows (*i.e. *genomic loci) were retained if they fell within 1 SD of the average for each of the three distributions, yielding a total of 164,884 loci. Di-nucleotide composition values for these sequences showed relatively high variances at individual di-nucleotides owing to the fact that there are only 149 di-nucleotide observations for the 16 possible di-nucleotides in each 150 bp window. Thus, we further reduced the set of sequences to loci that more closely match the pre-miRNA di-nucleotide composition. To do this, di-nucleotide compositions for each loci were represented as ordered vectors and a similarly ordered vector for average di-nucleotide values of the 715 pre-miRNAs was computed. Genomic loci di-nucleotide vectors were then compared to the pre-miRNA di-nucleotide vector using the Manhattan distance to select the 75% of loci most similar to the pre-miRNAs in di-nucleotide composition, yielding a total of 124,100 loci. From these loci, 1,000 random sets of 715 loci each (with the same length and size distribution as the pre-miRNAs) were taken as a collection of background control sets.

Functional analysis of TFs associated with TFBS located inside pre-miRs was carried out using GOStat [27], DAVID [28], and Fatigo [29]. Expression levels of miRNA genes across human tissues based on small RNA library sequencing were obtained from Liang and Li [30]. The conservation pattern of all human mature miRNAs across 44 vertebrate genomes was obtained from [6]. The evolutionary rate of miRNA genes was taken from [10] with additional data provided by Dr. Q. Cui. Most statistical analyses were performed using Chi-square tests. Analyses of dinucleotide composition and evolutionary rate of pre-miR sequences were performed using unpaired t-tests, 2-tailed.

### Analysis of all TFBS hits within all human pre-miRs

To analyze all TFBS motif hits regardless of whether they are conserved across species (Figure 3, 5 and 6), *Homo sapiens *pre-miR sequences were directly scanned for enrichment of transcription factor binding motifs. *Homo sapiens *pre-miR sequences were extracted from the miRBase 14 hairpin.fa FASTA sequence file [25]. Transcription factor binding site motifs and motif classes were acquired from the TRANSFAC 7.0 Public database [31]. The position frequency matrices were converted to position weight matrices using the TFBS::Matrix BioPerl module [32] and miRNA hairpin sequence were scanned for binding sites with the MOODS algorithm [33]. The transcription factor binding site motifs were limited to the 258 motifs included on the UCSC HMR Conserved Transcription Factor Binding Site track, and equivalent thresholds were maintained ([26] and Weirauch, M. personal communication). Of a total of 721 miRNA hairpins, 261 were considered primate specific and 460 were not ([6], as defined in their supplementary materials, using their more highly stringent definition of "primate specific"). For each pre-miR sequence, a first-order Markov transition matrix was built for each pre-miR and was used to generate 1,000 random nucleotide sequences of equivalent length and dinucleotide sequence composition. The number of motif hits was scored for each pre-miR sequence as well as for each of its randomized sequences. The observed motif hit counts upon the pre-miR set (at a z-score ≥ 1.64 or ≥ 2.33) was compared to the mean number of hits upon the corresponding negative control set; the mean and standard deviation of the negative control set followed a Gaussian distribution and was used to calculate the p-value (1-tailed test).

## List of Abbreviations

miRNA: microRNA; Pre-miR: Small hairpin microRNA precursor; Pri-miR: primary microRNA gene transcript; TFBS: transcription factor binding site.

## Competing interests

The authors declare that they have no competing interests.

## Authors' contributions

NS conceived of the study, and NS, IKJ and JP jointly designed the analyses. JP, AC and TR carried out the computational and statistical analyses. All authors helped to draft the manuscript, and all have read and approved the final manuscript.

## Reviewers' comments

Reviewer #1, Dr. Guillaume Bourque, McGill University, nominated by Dr. Jerzy Jurka, had the following comments:

This is an interesting paper that reports an over-representation of conserved TF binding motifs embedded in microRNA precursor sequences. Although this observation is not totally novel (see comment #1 below), the analysis is more comprehensive and the simulations designed to test the significance of this observation are non-trivial. One weakness of the paper in its current form is that it uses too many tables (there are 9) when I think that a few figures (there is currently only 1) would drive some of the points much better (see comment #2).

### Comments

#1 I didn't see a reference to the paper "Genomic analysis of human microRNA transcripts", Saini et al. PNAS 2007 which should be cited. The Figure 2 of that paper in particular is very similar to the main result of the current paper. You should explain how your work differs and expands on what was done previously.

Response: If you look closely at Figure 2 of the Saini et al paper, you will see that they characterized the regions UPSTREAM (+) and DOWNSTREAM (-) of the pre-miR sequence but they did NOT examine the pre-miR sequence itself! Nowhere in that paper do they demonstrate or even suggest the possibility that TF binding sites may reside within the pre-miR. However, we will add Saini et al to our reference list as providing prior supporting evidence for our own data showing that the regions immediately flanking the pre-miR are also enriched in TF binding sites (albeit to a lesser extent compared to within the pre-miR itself).

#2 There are many tables some with too little information (e.g. Table 3, Table 8), some with information that would be best represented by a figure (e.g. Table 7) and some with too much information that's not directly relevant to the main point (e.g. Table 9). I believe that many of these tables could be replaced by a few multi-panel figures (e.g. Table 3, 4) that would greatly enhance the readability of the paper.

Response: We have now represented several of the tables by figures. Notably, we simplified the presentation of Table 1 and converted it to a figure (Figure 2) to make it more readable. We also reorganized and simplified some of the text throughout the paper to increase the readability.

#3 One of the first questions I had when I read the first section of the result section (e.g. on page 5) was whether the observation made for precursor sequences was restricted to the actual precursor sequences or extended to the flanking regions.

Could you show this directly in Table 1 (now Figure 2) or, even better, in a figure? I know that you talk about these things later in a different section on the properties of pre-mirRNAs with motifs (page 7, par 2) but to me this goes earlier when you're trying to establish the association. Also, instead of Additional file 2, I think that a figure that shows where the motifs are relative to the precursors sequences and that the enrichment doesn't extend beyond those sequences would probably help significantly.

Response: These comments seem to imply that we are claiming that the TF binding sites are restricted to pre-miR sequences and NOT also enriched in flanking regions. However, as stated above, the enrichment does cover both the pre-miR and to a lesser extent, the flanking regions as well.

#4 Also about Table 1 (now Figure 2) and the enrichment, could you also include another control such as gene promoter sequences so that we can see the strength of the enrichment relative to a positive control?

Response: We appreciate the sentiment behind this request, but there are several problems with doing so. First, promoter sequences were used in the construction of the statistical model that defined motif matching and significance, so there is some circularity in using similar sequences for statistical testing. Second, the outcome of such a test is irrelevant to the point of our paper - it does not matter if the density of TF binding sites within pre-miRs is as great, greater than or less than the density within promoters. The fact that they are there AT ALL (much less in the majority of conserved pre-miRs) is surprising, unexpected and deserves to be acknowledged.

#5 Page 6, paragraph 2: Isn't this observation circular? You've looked for pre-miRNA sequences with conserved TFBS and you now observed that they are more conserved on a sequence-level... Wouldn't you have to look for any TFBS (whether conserved or not) and try to make that case?

Response: To some extent, what you are saying is true. However, the pre-miR sequences of highly conserved mature miRNAs do show significant drift in certain regions (e.g. the loop region). Since we showed that the TFBS sites are generally NOT co-located exactly with the mature miRNA sequence (Table 7, now Figure 4), there is no reason to assume that the set of conserved pre-miRs [defined by overall similarity across rat, mouse and human] should show the detailed conservation of exact TFBS motifs that it does, nor that it should extend to other vertebrate classes. More importantly, we show in a separate analysis that TFBS are highly enriched in pre-miRs even when the analysis includes all non-conserved sites and non-conserved pre-miRs. This analysis also shows that the prevalence for TFBS is greater in conserved pre-miRs than in primate-specific pre-miRs.

#6 Page 7, paragraph 1: Are the cancer pathways enriched for these miRNAs? If not this is not really a critical observation.

Response: Correct. The point is not that they are enriched in cancer miRs, but that they affect many of the most-studied miRs and pathways that investigators care about.

#7 Page 12, par 1 and Page 21, Table 1: "TFBS with experimental support", why do you mean here by experimental support? Do you mean that the motifs are experimentally supported? What is the source of the other ones? That wasn't clear to me. Also in that table, what are the two numbers in each cell? Average and St Dev?

This needs to be explained in the table caption. Do you mean 715 sets of 1000 sequences or 1000 set of 715 sequences (since that's the number of human pre-miRNAs that you use).

Response: We have simplified Table 1, changed it to a figure (Figure 2), and rewritten the legend so that it is now clear. We removed the separate data for "with experimental support" as not being essential.

#8 Page 22, Table 2 (now Figure 3): The enrichment is more subtle based on this test (not even 2 fold). Can you comment on this discrepancy in the discussion?

Response: There is no discrepancy here. In this case, we are examining all pre-miR sequences fully, rather than only conserved regions, so both the true hits and the baseline "noise" level of hits are higher than when only conserved hits were considered. For example, on the top line of Table 2 (now Figure 3), the average number of TFBS hits in the randomized set is 4016 with a SD of 97. Stated another way, the null distribution of hits expected by chance has a mean of 4016 and SD of 97. What we actually observed in human pre-miRs is an average of 4721 hits. 4721-4016 = 705, which means the observed value is 7.268 SD away from the mean of the null distribution. This is extremely unlikely to have occurred by chance. What is important is the difference between pre-miRs and randomized pre-miR sequences, in terms of Standard Deviations - not the fold difference in hits.

### Small comments

Page 3, par 2, line 1: "track is visible" - > "track is available"

Page 3, par 2, line 3: "398 transcription factor binding sites", this is a bit confusing to me. Do you mean 398 transcription factor binding motifs? The term "binding site" is used to describe a specific instance of a binding motif.

Response: Done.

Page 10, par 2, line 11: "Importantly, since this paper was originally submitted for publication, Zhu et al have reported" - > "Consistent with our findings, Zhu et al. have recently reported"

Response: This erroneously implies that their observations predated ours.

Reviewer #2, Dr. Dmitri Pervouchine, Moscow State University, nominated by Dr. Mikhail Gelfand, had the following comments:

In order to check whether the reported association is indeed present, I sampled 20 human microRNAs and looked them up by eye in the Genome Browser. Of these, 16 cases were not associated with TRANSFAC-predicted binding sites.

Response: Is the reviewer saying that out of 20 human pre-miRs which we claimed to have TFBS, 16 were not supported by eye in the Genome Browser? That would indicate a serious problem with our ms. and we would appreciate clarification of this point. However, it seems that he merely chose 20 in an unsystematic manner. Many human miRs are primate specific and will not show TFBS in the Genome Browser.

hsa-mir-17 belonged to a polycistronic cluster (also containing hsa-mir-18a, hsa-mir-19a, and hsa-mir-20a) residing in a large genomic region highly enriched with TF binding sites, let-7a and let-7f, also likely to be transcriptionally coupled, were also enriched with TFBSs, and mir-7-1 was also found in a large genomic region with high density of TFBSs. In this regard one should ask whether or not miRNAs tend to occur in genomic loci with higher than on average TFBS density (this is different from the statement made in the paper).

Response: As discussed above with regard to the comments of reviewer 1, TFBS motifs are indeed enriched in regions flanking pre-miRs [that was previously known] as well as within pre-miRs [our novel observation].

The authors should make a statistical control by using genomic regions with high overall TFBS density to address the possible confounding effect.

Response: We did that. They comprise the negative control dataset comprised of sequences "most similar" to pre-miRs in conservation and dinucleotide sequence composition (results shown in Figure 2).

Another statistical control comparing to hairpins that are similar to microRNAs would be necessary to address whether or not the RNA structure is responsible for the seeming relationship.

Response: We agree that it is likely that the association of TFBS motifs is related somehow to the hairpin structure of pre-miRs. However, were that to be true [and to hold for some other miR-like hairpins in the genome], it would only make our data more interesting and provide more biological context (e.g., it might tie in with the observation that some transcription factors bind double-stranded sequences). It would not imply that our observations are some type of artifact. One might think of snoRNAs as a putative negative set, but we now know that many snoRNAs actually give rise to miRNA-like small RNAs which may be functionally related to miRNAs. Thus, it is not clear whether snoRNAs should be appropriately viewed as NEGATIVE control sequences, or potentially as additional POSITIVE examples! In short, we do not know of any dataset of "hairpins similar to microRNAs" that should definitely be negative and that can be used unambiguously for such a test.

Also, another control would be necessary to address to what extent the observed association is influenced by the cluster organization of miRNAs.

Response: We did that. As shown in Table 3 (now Table 1), the phenomenon affects clustered and unclustered miRs equally.

Accordingly, the manuscript "Transcription factor binding sites are highly enriched within microRNA precursor sequences" in its current form is not recommended for publication.

Response: The most important point of our paper is that the MAJORITY of conserved human pre-miRs express one or more transcription factor binding sites, as defined by the same algorithms and stringent statistical criteria that are used for TFBS within promoters. In our view, this is likely to have BIOLOGICAL significance regardless of the level of statistical significance. The fact that the statistical significance is also extremely high is a bonus. Had we reported the presence of TFBS just upstream of pre-miRs (as Saini et al did), no one would have questioned our observation in the slightest. It is only because current knowledge does not provide an obvious expectation that TFBS should be present, that we believe reviewers have had such strong objections to our paper. Yet, we feel that one of the major reasons for carrying out bioinformatics analyses is to make surprising observations that can stimulate further mechanistic investigations. The recent Zhu et al paper already lends further independent bioinformatics support to our observations, and we pointed out that the experimental literature offers two tentative biological explanations - namely, that pre-miRs contain promoter elements, and/or that transcription factors bind pri-miRs and pre-miRs directly. Thus, we believe that publication at this point is justified.

Reviewer #3, Dr. Yuriy Gusev, Georgetown University Medical Center, provided no comments for publication.

## Supplementary Material

Additional file 1 **Spreadsheet containing a list of all human pre-miRs that contain high-confidence conserved TFBS motifs (z-score ≥ 2.33) (sheet 1) and a list of TRANSFAC transcription factors that bind these TFBS motifs (sheet 2)**. Click here for file

Additional file 2 **File showing the location of each high-confidence conserved TFBS (z-score ≥ 2.33) on each pre-miR sequence**. Positions of miRBase-annotated mature miRNA sequences (OOOO....) and miRNA* sequences (NNNNN....) are indicated within each pre-miR. Click here for file
